# Eculizumab treatment alters the proteometabolome beyond the inhibition of complement

**DOI:** 10.1172/jci.insight.169135

**Published:** 2023-07-10

**Authors:** Christopher Nelke, Christina B. Schroeter, Frauke Stascheit, Niklas Huntemann, Marc Pawlitzki, Alice Willison, Saskia Räuber, Nico Melzer, Ute Distler, Stefan Tenzer, Kai Stühler, Andreas Roos, Andreas Meisel, Sven G. Meuth, Tobias Ruck

**Affiliations:** 1 Department of Neurology, Medical Faculty, Heinrich Heine University Düsseldorf, Düsseldorf, Germany.; 2 Charité – Universitätsmedizin Berlin, corporate member of Freie Universität Berlin and Humboldt Universität zu Berlin, Department of Neurology and Experimental Neurology, Berlin, Germany.; 3 Charité – Universitätsmedizin Berlin, corporate member of Freie Universität Berlin and Humboldt Universität zu Berlin, Neuroscience Clinical Research Center, Berlin, Germany.; 4 University Medical Center of the Johannes-Gutenberg University Mainz, Mainz, Germany.; 5 Helmholtz Institute for Translational Oncology (HI-TRON) Mainz, Germany.; 6 Deutsches Krebsforschungszentrum (DKFZ), Heidelberg, Germany.; 7 Molecular Proteomics Laboratory, Heinrich Heine University, Düsseldorf, Germany.; 8 Department of Neuropediatrics, University of Duisburg-Essen, Essen, Germany.; 9 Charité – Universitätsmedizin Berlin, corporate member of Freie Universität Berlin and Humboldt Universität zu Berlin, Center for Stroke Research Berlin, Berlin, Germany.

**Keywords:** Autoimmunity, Neuroscience, Autoimmune diseases, Complement, Neuromuscular disease

## Abstract

Therapeutic strategies targeting complement have revolutionized the treatment of myasthenia gravis (MG). However, a deeper understanding of complement modulation in the human system is required to improve treatment responses and identify off-target effects shaping long-term outcomes. For this reason, we studied a cohort of patients with MG treated with either eculizumab or azathioprine as well as treatment-naive patients using a combined proteomics and metabolomics approach. This strategy validated known effects of eculizumab on the terminal complement cascade. Beyond that, eculizumab modulated the serum proteometabolome as distinct pathways were altered in eculizumab-treated patients, including the oxidative stress response, mitogen-activated protein kinase signaling, and lipid metabolism with particular emphasis on arachidonic acid signaling. We detected reduced levels of arachidonate 5-lipoxygenase (ALOX5) and leukotriene A_4_ in eculizumab-treated patients. Mechanistically, ligation of the C5a receptor (C5aR) is needed for ALOX5 metabolism and generation of downstream leukotrienes. As eculizumab prevents cleavage of C5 into C5a, decreased engagement of C5aR may inhibit ALOX5-mediated synthesis of pro-inflammatory leukotrienes. These findings indicate distinct off-target effects induced by eculizumab, illuminating potential mechanisms of action that may be harnessed to improve treatment outcomes.

## Graphical abstract

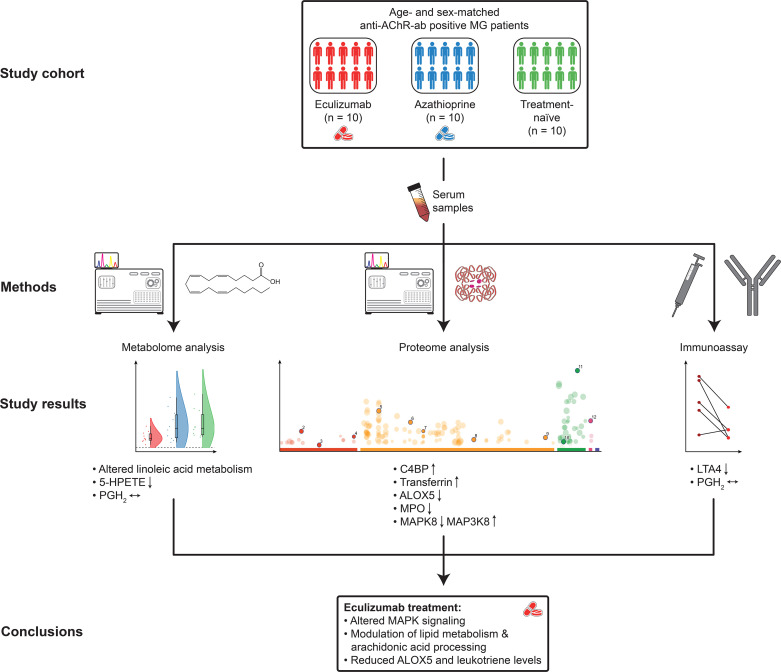

## Introduction

Immunotherapies have long been used to treat autoimmunity. With the advent of targeted immunotherapies, a new generation of treatment options emerged for autoimmune diseases. These agents may provide sustained immune modulation with a more beneficial adverse effect profile than traditional immunosuppressants (1). One of these agents is the monoclonal antibody (Ab) eculizumab used to treat complement-mediated diseases (2, 3). Eculizumab specifically binds to complement protein C5, preventing its cleavage into C5a and C5b, thereby precluding the formation of the terminal complement cascade (4). While eculizumab has demonstrated high efficacy even in cases of treatment-refractory autoimmune disease (2, 5, 6), studies investigating the long-term safety and mechanistic consequences are needed (7). Even more recently, a number of C5 inhibitors were developed, including ravulizumab, a monoclonal Ab with enhanced effect duration (8), and zilucoplan, a small molecule inhibitor of C5 (9), among others, which have revolutionized the treatment of several autoimmune disorders. This trend underlines the need for a deeper understanding of terminal complement inhibition. Following this line of thought, novel functions for complement have emerged besides its critical role in innate immunity, including regulation of effector T cell differentiation and of metabolic pathways (10, 11). Consequently, therapeutic ablation of the terminal complement cascade is likely to influence human biology beyond innate immune response inhibition.

Thus, we employed an in-depth proteomics and metabolomics approach to dissect the serological profile of anti–acetylcholine receptor antibody–positive (anti-AChR Ab^+^) myasthenia gravis (MG) patients treated with eculizumab. Insight into the advantageous and disadvantageous consequences of treatment may prove valuable for patient selection and development of risk mitigation strategies.

## Results

### Eculizumab affects the terminal complement cascade.

**Figure 1:**
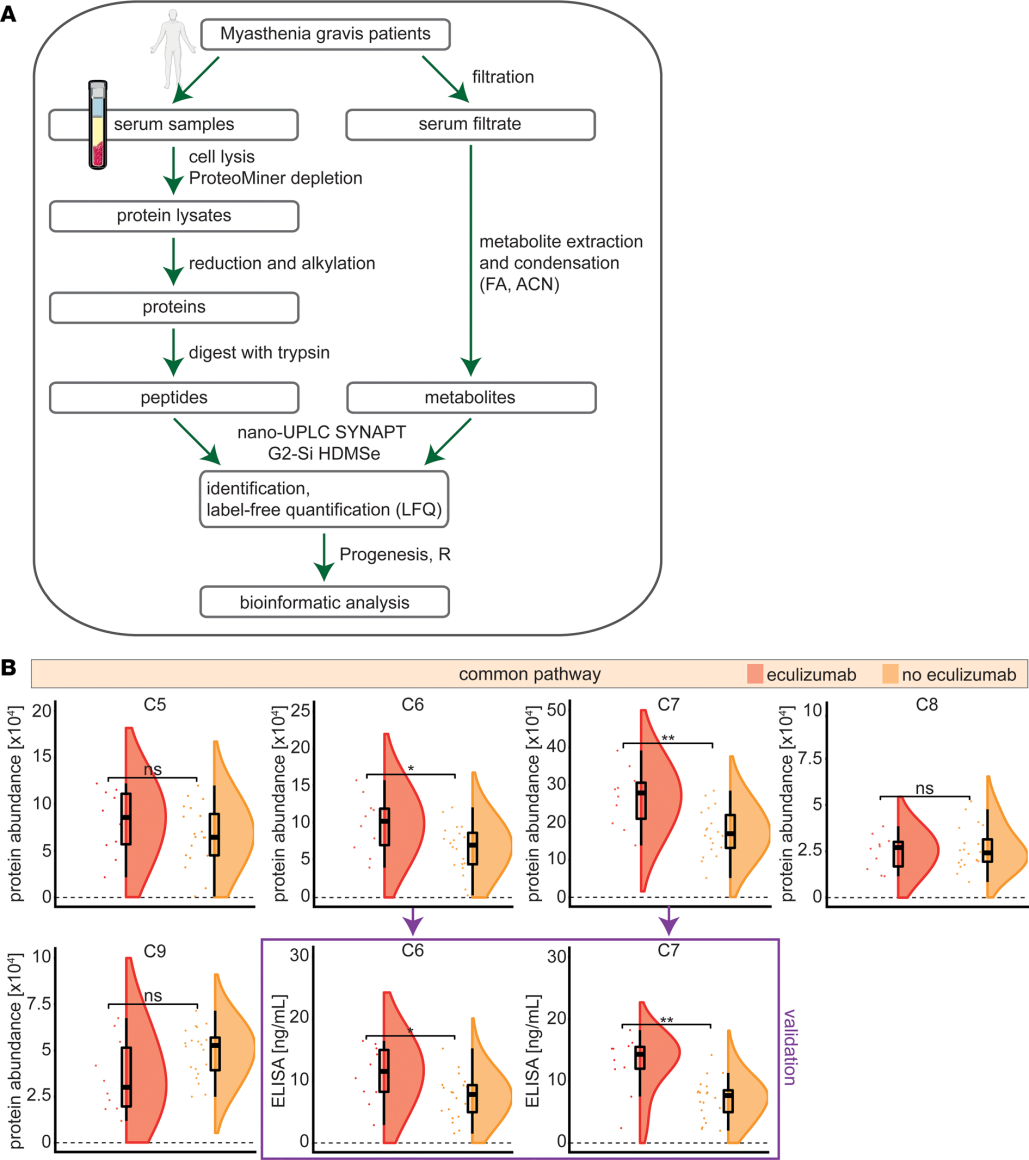
Eculizumab inhibits the terminal complement cascade. (**A**) Schematic overview of workflow. Mass spectrometry–based proteomic and metabolomic analysis of serum samples was performed using a label-free, unbiased approach and nano-UPLC HDMSe (SYNAPT G2-Si). (**B**) Protein abundance of serum proteins determined by proteomics displayed as raincloud plots. Protein levels were determined by ELISA measurement for C6 and C7 as validation. Whiskers are 1.5 IQR. Differences were determined by the 2-sided Student’s *t* test for eculizumab (red) (*n* = 10) and no eculizumab (orange) (*n* = 20). ACN, acetonitrile; FA, formic acid; HDMSe, high-definition mass spectrometry; UPLC, ultra-performance liquid chromatography. **P < 0.01, *P < 0.05.

Aiming to explore the influence of eculizumab on the serum proteome and metabolome, we included 3 age- and sex-matched cohorts of patients with MG, treated with eculizumab or azathioprine (as most common first-line therapy) and treatment-naive patients (as control group). Patients were approximately 55 years old at baseline, with 2 women and 8 men in each group (Table 1). Eculizumab-treated patients received 2.8 (SD 1.6) previous ISTs, while azathioprine-treated patients received 1.5 (SD 2.5). Thymectomy had been performed in 3 eculizumab-treated, 1 treatment-naive, and 5 azathioprine-treated patients. Thymectomy was performed at least 6 months before study inclusion for all patients. No differences in steroid dosage were observed between eculizumab- and azathioprine-treated patients (*P* = 0.68). We performed in-depth proteomic analysis of patient serum samples using shotgun mass spectrometry (Figure 1A). To underline the feasibility of a proteomics approach, we first analyzed the common complement cascade of patients treated with eculizumab compared with all other patients. Complement proteins were readily detected (Figure 1B). Eculizumab forms a complex with C5 precluding the latter from cleavage, thus reducing the levels of free C5 in serum (12). Following lysis for proteomic analysis, the eculizumab-C5 complex was broken down and the individual peptides were recorded. Consequently, C5 levels were unchanged when measured by proteomics as compared to ELISA (Supplemental Figure 4A; supplemental material available online with this article; https://doi.org/10.1172/jci.insight.169135DS1) (12). In line with the inhibitory effect of eculizumab on C5 cleavage, proteomics detected increased levels of C6 and C7. To validate these results, we performed ELISA studies of C6 and C7 levels and verified that these proteins were increased in eculizumab-treated patients (Figure 1B). Intriguingly, while downstream complement factors C6 and C7 accumulated in eculizumab-treated patients, levels of C8 and C9 remained unchanged.

Taken together, analysis of the serum proteome corroborates eculizumab’s mechanism of action.

### Eculizumab alters the serum proteome beyond complement inhibition.

**Figure 2:**
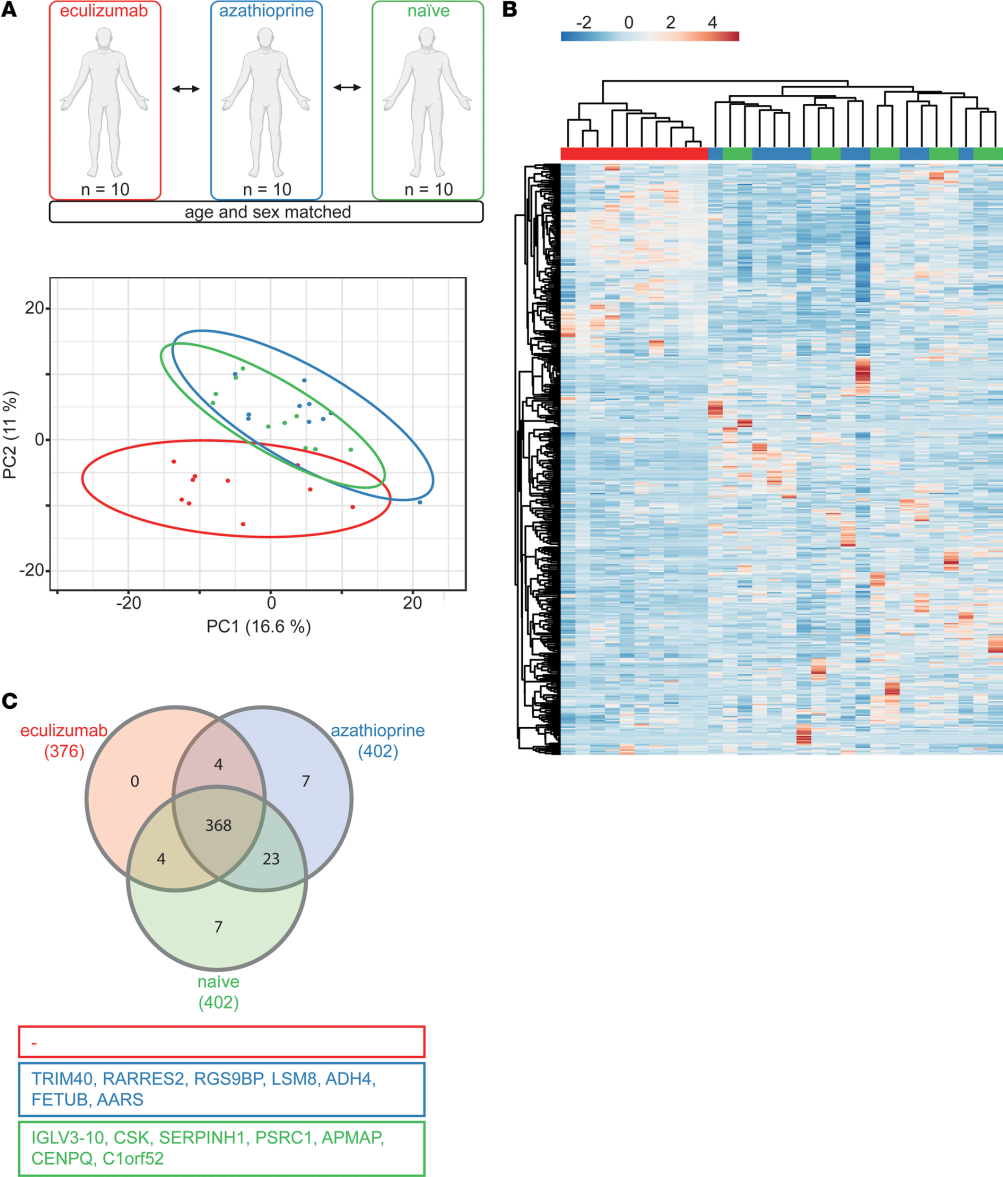
Eculizumab shapes the serum proteome. (**A**) PCA of serum proteome. SVD was used to calculate principal components. The *x* and *y* axes show principal component 1 and principal component 2, respectively. Prediction ellipses are such that with probability 0.95, a new observation from the same group will fall inside the ellipse. (**B**) Heatmap of serum proteome. Rows (proteins) and columns (replicates, here: patients) were clustered using correlation distance and average linkage. For multivariate analysis, rows were centered and unit variance scaling was applied. *P* value for significance was set to <0.05. (**C**) Venn diagram of serum proteome showing overlapping as well as unique expression profiles of the 3 analyzed data sets (*n* = 10 for each group). *N* = 10 per group. PCA, principal component analysis; SVD, singular value decomposition.

Next, we assessed changes to the serum proteome eculizumab treatment induced. To this end, we reduced data set complexity by principal component analysis (PCA) (Figure 2A). Here, azathioprine-treated and treatment-naive patients were indistinguishable, while eculizumab-treated patients displayed altered clustering. Consistent with this, superimposing hierarchical clustering on the heatmap visualization separated eculizumab patients from all others (Figure 2B). Aside from shared protein patterns, we analyzed unique proteins for each cohort. As displayed in Figure 2C, the azathioprine and treatment-naive cohorts were characterized by 7 unique proteins. In contrast, the eculizumab cohort demonstrated no unique proteins.

**Figure 3:**
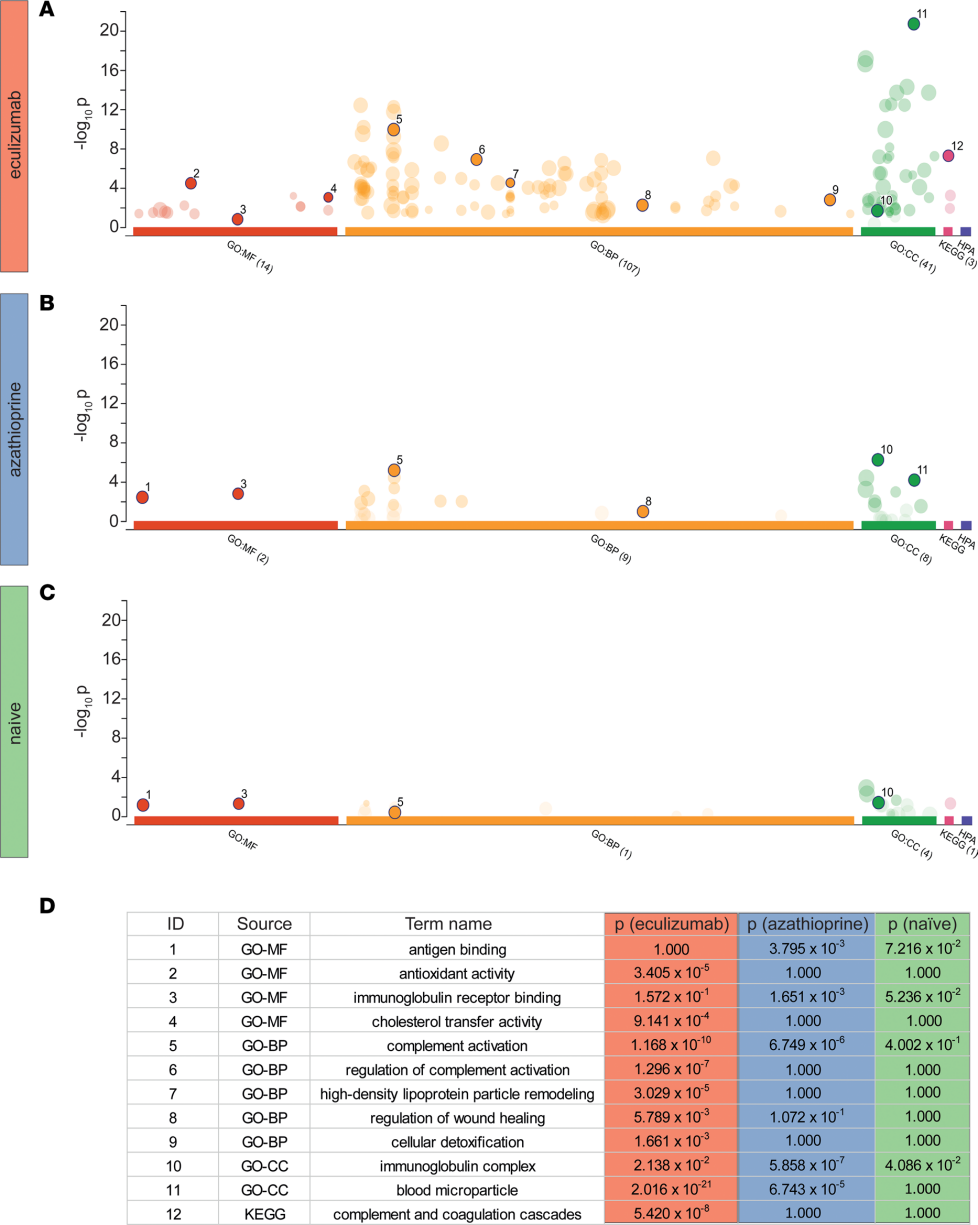
Terminal complement inhibition influences functional pathways. (**A**–**C**) Manhattan plot illustrating functional enrichment of differentially regulated proteins (fold-change > 1.5) from the MF (molecular function), BP (biological process), CC (cellular component), KEGG (Kyoto Encyclopedia of Genes and Genomes), and HPA (Human Protein Atlas) databases, respectively. The analysis was performed using the R package gprofiler2 with g:SCS multiple-testing correction method applying significance threshold of 0.05. The –log_10_ *P* value is indicated on the *y* axis. IDs next to circles are annotated in **D**. Related GO terms and functional pathways were clustered together. The number in parentheses behind all depicted GO terms on the *x* axis denotes the number of found GO terms and functional pathways. (**D**) Table displaying GO terms and functional pathways of interest shown in **A**–**C**. *N* = 10 per group. GO, gene ontology.

To further dissect the proteome patterns resulting from eculizumab treatment, we performed functional enrichment analyses of differentially expressed proteins. Here, eculizumab treatment showed marked alterations of Gene Ontology (GO) terms and functional pathways when compared with azathioprine or treatment-naive patients (Figure 3, A and D). For MFs, the eculizumab cohort was enriched for antioxidant activity and cholesterol transfer activity, among others. In contrast, antigen binding was significantly enriched in treatment-naive and azathioprine-treated patients’ serum samples but not in eculizumab-treated patients (Figure 3, B and C). For BPs, we found functional alterations for complement activation and regulation of complement activation in the eculizumab cohort, likely due to accumulation of terminal complement factors. Interestingly, serum from eculizumab-treated patients also revealed significantly altered high-density lipoprotein particle remodeling, regulation of wound healing, and cellular detoxification processes compared with azathioprine or treatment-naive patients. For CCs, we observed GO term enrichment for immunoglobulin complexes and blood microparticles in the eculizumab group, among others. Functional analysis of the KEGG pathway showed upregulation of complement and coagulation cascades in the eculizumab cohort. Analysis of the HPA database revealed no significant functional enrichment.

Together, these findings point toward highly complex consequences of eculizumab treatment, with alterations in multiple biological pathways that go beyond complement inhibition.

### Distinct immunometabolic pathways are altered in eculizumab-treated patients.

**Figure 4:**
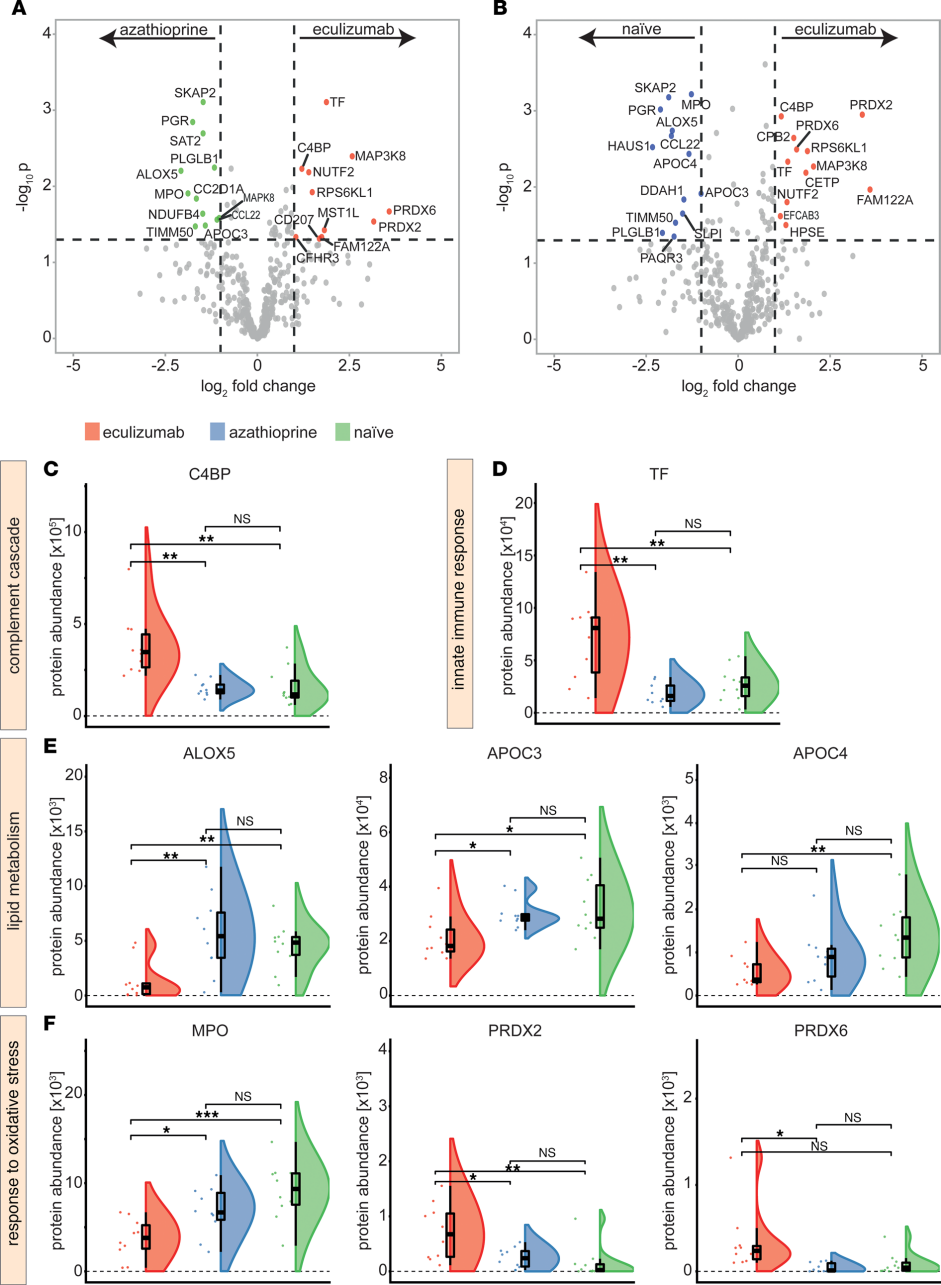
Unique protein signatures are observed for eculizumab-treated patients. (**A**) Volcano plot comparing azathioprine and eculizumab constructed by plotting log_2_ values of protein fold-changes between the 2 treatment groups against their –log_10_ *P* values. Differential regulation of expression levels was calculated by 1-way ANOVA. The horizontal dashed line indicates the significance cutoff value, which was set to *P* < 0.05. The vertical dashed lines indicate a fold-change cut-off of 1.0. (**B**) Volcano plot comparing treatment-naive and eculizumab-treated patients. (**C**–**F**) Protein abundance of significantly regulated serum proteins of interest in **A** displayed as raincloud plots. Whiskers are 1.5 IQR. To account for multiple comparisons, statistical significance was corrected by the false discovery rate (FDR) approach. A threshold of *q* = 5% was used for FDR. One-way ANOVA testing was used for comparison of multiple groups. *N* = 10 per group. ALOX5, arachidonat-5-lipoxygenase; APOC3/4, apolipoprotein CIII/IV; C4BP, C4b-binding protein; CRP, C-reactive protein; MPO, myeloperoxidase; PRDX2/6, peroxiredoxin-2/6; TF, transferrin. ***P < 0.001, **P < 0.01, *P < 0.05.

**Figure 5:**
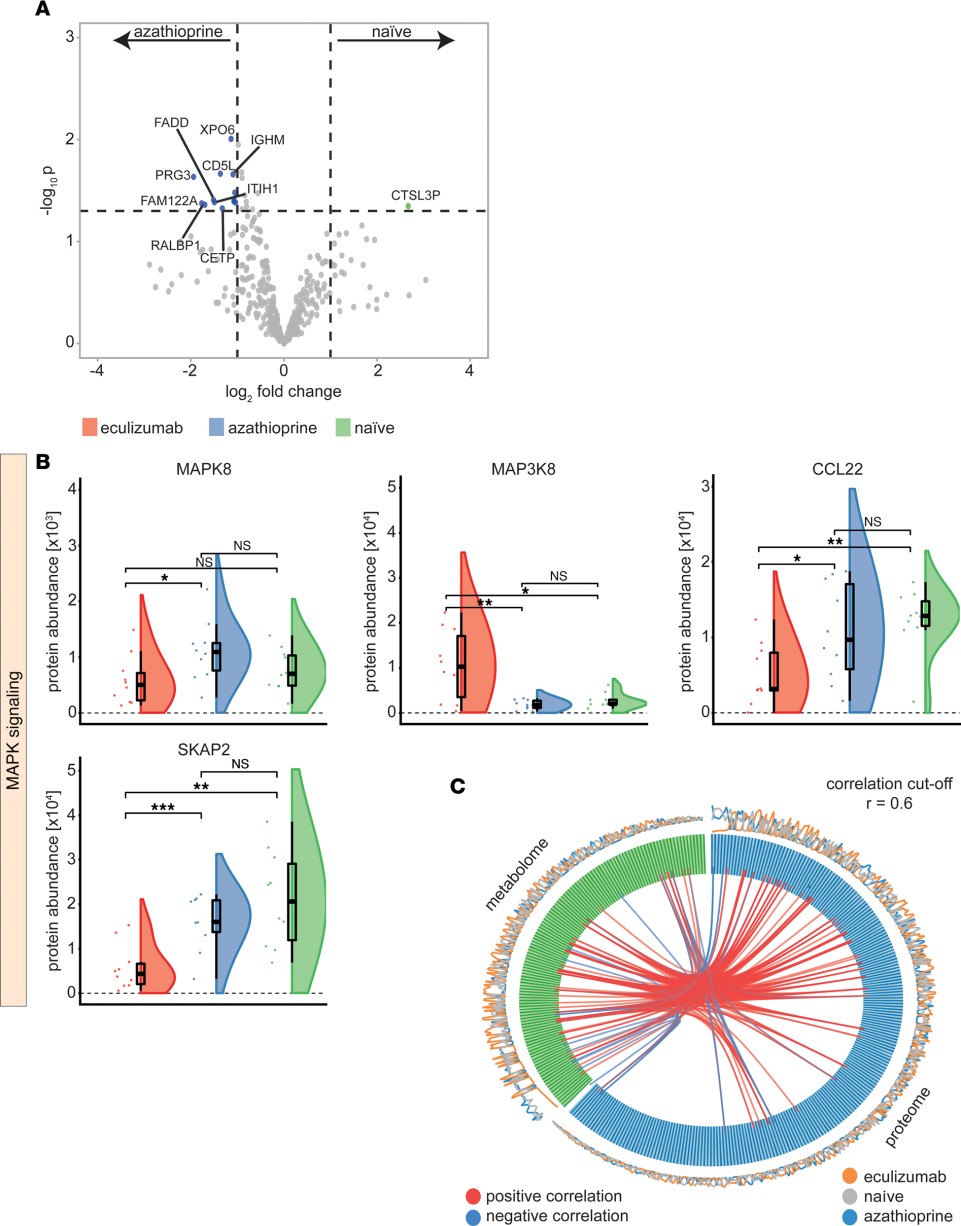
Eculizumab-associated changes to the proteometabolome. (**A**) Volcano plot comparing azathioprine and treatment-naive patients constructed by plotting log_2_ values of protein fold-changes between the 2 treatment groups against their –log_10_ *P* values. Differential regulation of expression levels was calculated by 1-way ANOVA. The horizontal dashed line indicates the significance cutoff value, which was set to *P* < 0.05. The vertical dashed lines indicate a fold-change cutoff of 1.0. (**B**) Protein abundance of significantly regulated serum proteins of interest displayed as raincloud plots. Whiskers are 1.5 IQR. To account for multiple comparisons, statistical significance was corrected by the false discovery rate (FDR) approach. A threshold of *q* = 5% was used for FDR. (**C**) Circos plot visualizing correlations between the proteome (blue) and metabolome (green). Correlations were calculated by pairwise variable association for large data matrixes. Inner lines display positive (red) and negative (blue) correlations between individual metabolites above the threshold of *r* = 0.6. The outer circle displays the serum abundance of individual data points measured by mass spectrometry. One-way ANOVA testing was used for comparison of multiple groups. *N* = 10 per group. CCL22, CC motif chemokine ligand 22; MAPK8, mitogen-activated protein kinase 8; MAP3K8, mitogen-activated protein kinase kinase kinase 8; PGR, progesterone receptor; SKAP2, Src kinase-associated phosphoprotein 2. ***P < 0.001, **P < 0.01, *P < 0.05.

Aiming to dissect proteome patterns induced by terminal complement ablation, we compared individual proteins between groups (Figure 4, A and B, and Figure 5A). Here, we observed a marked increase of C4BP in serum of eculizumab-treated patients as compared with the other groups (Figure 4C). Besides, TF was increased in eculizumab-treated patients (Figure 4D). To validate these findings with a different method, C4BP and TF levels were measured by ELISA, corroborating the observations of proteomic analysis (Supplemental Figure 4, A and B). Consistent with the patterns observed for the GO term analysis, ALOX5 and APOC3/4 were altered in eculizumab-treated patients, pointing toward changes of the lipid metabolism in response to terminal complement ablation (Figure 4E). ALOX5 levels were verified by ELISA measurement (Supplemental Figure 4C). A further pattern emerged for the oxidative stress response with the MPO being reduced in the eculizumab group, while the antioxidant enzymes PRDX2/6 were increased (PRDX2 was significantly increased only when compared with treatment-naive patients) (Figure 4F). MAPKs are a ubiquitous group of enzymes that orchestrate cellular responses to a wide range of stimuli, including pro-inflammatory cytokines or osmotic stress (13).

Interestingly, various members of this group were altered in serum of eculizumab-treated patients: levels of MAPK8 were decreased compared with the azathioprine cohort, while MAP3K8 levels were significantly increased (Figure 5B). MAP3K8 is of interest as it represents a promoter for production of TNF-α and IL-2 in T lymphocytes. SKAP2 is required for macrophage migration and actin reorganization (14). We detected reduced SKAP2 levels in eculizumab-treated patients compared with azathioprine patients or treatment-naive controls (Figure 5B). Concurrently, the chemokine CCL22 was reduced in eculizumab-treated patients as compared with controls. The change of CCL22 was validated by ELISA (Supplemental Figure 4D).

### Integration of metabolites indicates alterations of arachidonic acid metabolism by eculizumab.

**Figure 6:**
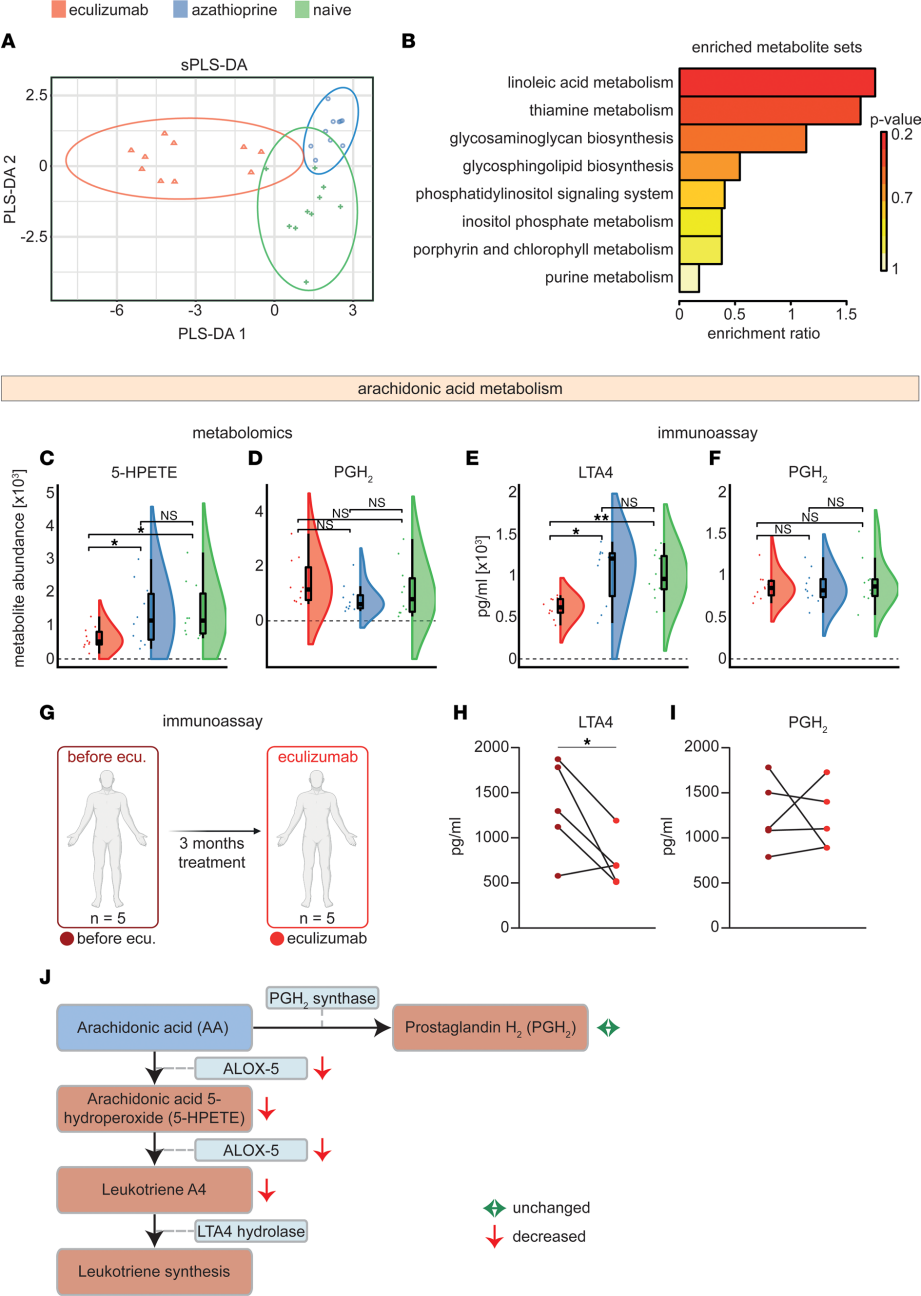
Immunometabolic pathways are altered in eculizumab-treated patients. (**A**) sPLS-DA score plot showing the metabolite data set for different groups. Prediction ellipses are 95% CI. (**B**) Functional enrichment analysis of the eculizumab cohort compared with other groups. Enrichment was performed by MetaboAnalystR package version 3.0 set to GSEA using the KEGG database. (**C** and **D**) Metabolite abundance of 5-HPETE and PGH_2_ displayed as raincloud plots. Metabolite levels were determined by metabolomic analysis. (**E** and **F**) Metabolite abundance of LTA_4_ and PGH_2_ displayed as raincloud plots. Metabolite levels were determined by immunoassay. Whiskers are 1.5 IQR. (**G**) For longitudinal analysis, samples were acquired from 5 patients before and under eculizumab treatment. (**H**) Longitudinal analysis shows reduction of LTA_4_ level in eculizumab-treated patients. (**I**) PGH_2_ levels remained unchanged after switching to eculizumab. “Before eculizumab” and “eculizumab” are represented in dark red and red, respectively. (**J**) Schematic overview of AA metabolism. Changes observed in this study are indicated by arrows. To account for multiple comparisons, statistical significance was corrected by the false discovery rate (FDR) approach. A threshold of *q* = 5% was used for FDR. ANOVA testing was used for comparison of multiple groups. *N* = 10 per group. 5-HPETE, arachidonic acid 5-hydroperoxide; GSEA, gene set enrichment analysis; LTA_4_, leukotriene A_4_; PGH_2_, prostaglandin H_2_; sPLS-DA, sparse partial least squares discriminant analysis.

Next, we analyzed our metabolome data set, aiming to elaborate observed patterns following complement treatment. After data pre-processing and metabolite identification, a total of 289 unique metabolites were permitted for downstream analysis. Given the ability of sparse partial least squares discriminant analysis (sPLS-DA) to analyze noisy data with high collinearity (15), we tuned a model to fit our metabolomics data and reduce data set dimensionality (Figure 6A and Supplemental Figure 1). Here, all 3 groups demonstrated individual metabolome signatures. To identify altered pathways, we performed functional enrichment analysis of the eculizumab cohort using the KEGG database (Figure 6B). Interestingly, linoleic acid metabolism was the most enriched functional pathway in the eculizumab cohort. Given our previous observation of reduced ALOX5 levels, we suspected that arachidonic acid (AA) metabolism is altered due to complement blockade in eculizumab-treated patients. Linoleic acid is a precursor lipid metabolized to AA. Thus, we investigated metabolites correlating with ALOX5 by constructing a circos plot using the mixOmicsR package as adapted from González et al. (16) (Figure 5C). 5-HPETE was positively correlated with ALOX5. As such, we hypothesized that reduced ALOX5 levels result in altered AA metabolism and inhibition of downstream production of leukotrienes (17). AA is metabolized by ALOX5, resulting in 5-HPETE, which is further processed to LTA_4_ (17). Indeed, comparison between groups revealed that 5-HPETE was reduced in the serum of eculizumab patients (Figure 6C). 5-HPETE is metabolized to LTA_4_, a precursor of downstream leukotriene synthesis (18). However, LTA_4_ could not be unambiguously detected in our metabolomics approach. In addition to its role in the leukotriene pathway, AA is also a key precursor of prostaglandins. To investigate whether prostaglandin metabolism is also disturbed, we investigated levels of PGH_2_ resulting from enzymatic activity of the PGH_2_ synthase (Figure 6D). PGH_2_ levels were unchanged across groups. Finally, we aimed to corroborate our previous data in a different methodical readout. To this end, we performed targeted analysis of PGH_2_ and LTA_4_ by ELISA. We analyzed these metabolites as they constitute important precursor metabolites for both prostaglandin and leukotriene metabolism. Here, LTA_4_ levels were diminished in eculizumab-treated patients, while PGH_2_ was unaltered across groups (Figure 6, E and F). To validate these observations, we acquired longitudinal samples from eculizumab-treated patients. We collected serum samples from 5 patients treated with azathioprine who were switched to eculizumab (Figure 6G). These patients were on average 52.5 years old (range 31 to 73) with 3 men and 2 women (Table 2). After the switch, azathioprine was discontinued, patients were kept on eculizumab for 3 months, and serum samples were collected. Glucocorticoid dosages were kept stable for all patients with an average of 5 mg (SD 2.5). We measured LTA_4_ and PGH_2_ levels by ELISA comparing the status under treatment with eculizumab and before. Here, LTA_4_ was decreased in eculizumab-treated patients, while PGH_2_ levels remained unchanged (Figure 6, H and I).

Our data suggest that terminal complement blockade induces distinct alterations of AA homeostasis with inhibition of leukotriene production via ALOX5 metabolism (Figure 6J).

### Interaction of C5a with the C5a receptor is needed for AA processing by ALOX5.

**Figure 7:**
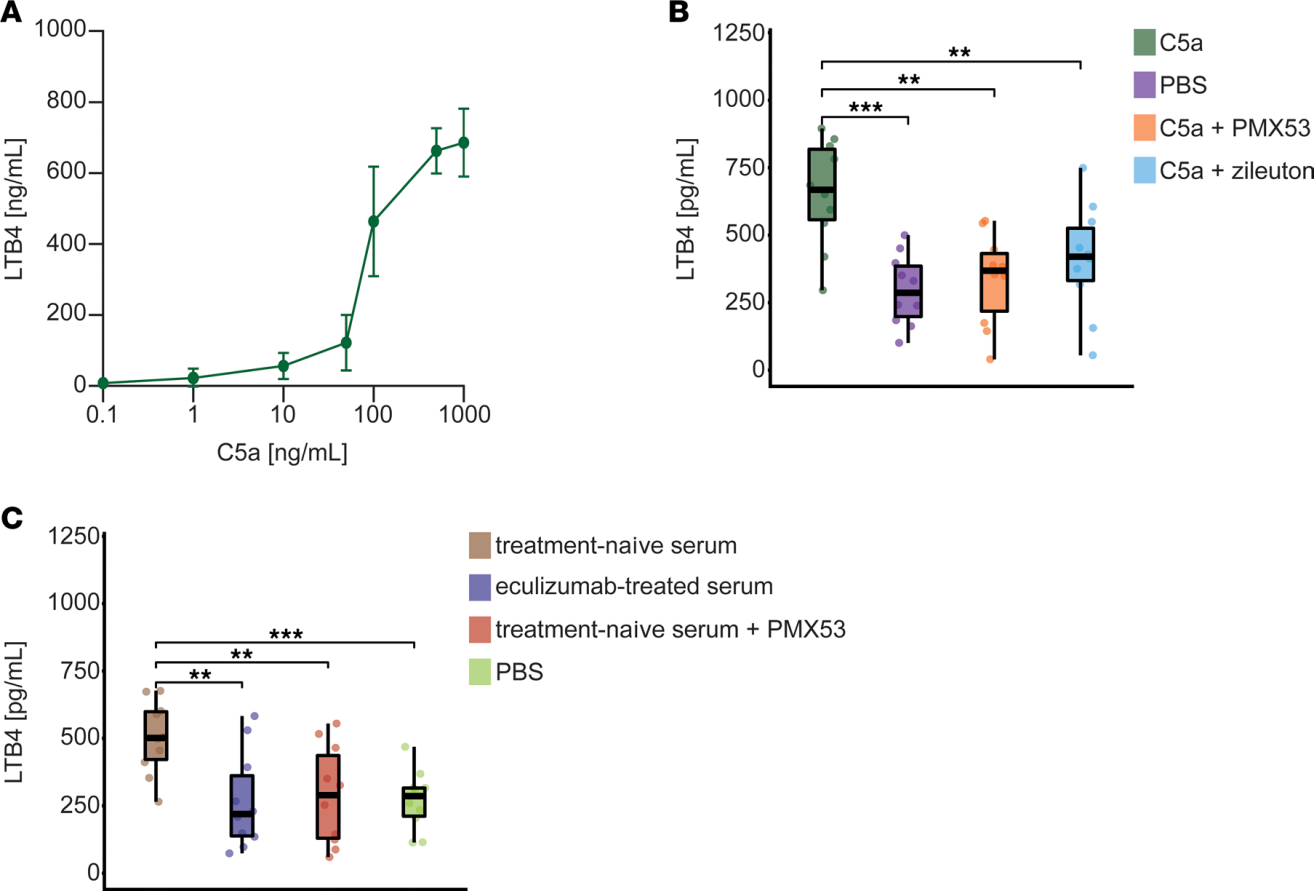
Interaction of C5a with the C5aR is needed for AA processing by ALOX5. (**A**) PMNs were incubated with indicated concentrations of recombinant C5a for 6 hours. Concentrations of LTB_4_ were determined by ELISA (*n* = 5). (**B**) PMNs were incubated with indicated substances (C5a 500 ng/mL, C5a + PMX53 10 nM, C5a + zileuton 100 μM, or PBS) for 6 hours (*n* = 10 per group). (**C**) PMNs were incubated with indicated serum from treatment-naive patients with MG or eculizumab-treated patients with MG for 6 hours. PMNs were also incubated with treatment-naive serum and a final concentration of PMX53 of 10 nM or PBS for 6 hours (*n* = 10 per group). Box plots show the interquartile range (box), median (line), and minimum and maximum (whiskers). Groups were compared by 2-sided Student’s *t* test. ***P < 0.001, **P < 0.01. ALOX5, arachidonate 5-lipoxygenase; LTB_4_, leukotriene B_4_; PMNs, polymorphonuclear leukocytes.

Last, we aimed to understand the mechanistic link between terminal complement inhibition and altered ALOX5 metabolism. Two previous studies demonstrated that ligation of the C5a receptor (C5aR) induces synthesis and release of leukotriene B_4_ (LTB_4_) (19, 20). LTB_4_ is a downstream product of AA and LTA_4_ exhibiting pro-inflammatory properties. As such, ALOX5 processing is required for LTB_4_ generation (19, 20). We suspected that reduced engagement of the C5aR downregulates ALOX5 activity in response to C5 inhibition, thereby decreasing LTA_4_ and LTB_4_ levels. To test this hypothesis, we investigated an in vitro model employing polymorphonuclear leukocytes (PMNs), the immune cells primarily involved in ALOX5 metabolism (21, 22). To validate the ability of ALOX5 to produce LTB_4_ in PMNs, we incubated these cells with increasing concentrations of recombinant C5a (Figure 7A). LTB_4_ was quantified using an ELISA. In line with previous reports (19, 20), C5a induced LTB_4_ release in a dose-dependent manner. Based on these observations, we chose 500 ng/mL of C5a for further experiments. Next, we wanted to demonstrate that C5aR is required for LTB_4_ release on PMNs. For this purpose, PMNs were incubated with C5a as well as PBS as control (Figure 7B). We used the C5aR inhibitor PMX53 (23) to test if the interaction of C5a and C5aR is needed for LTB_4_ generation. Indeed, co-incubation with PMX53 (10 nM) diminished LTB_4_ release. Second, PMNs were incubated with C5a and the ALOX5 inhibitor zileuton (24) (100 μM), also resulting in reduced LTB_4_ levels. Taken together, these data suggest that interaction of C5a and C5aR triggers leukotriene synthesis by ALOX5.

Next, as the C5 inhibitor eculizumab prevents cleavage of C5 and release of C5a, we suspect that this mechanism is responsible for the observed effects. To test this hypothesis, we incubated PMNs with the serum of the 10 treatment-naive patients employed in this study as well as serum from the eculizumab-treated patients (Figure 7C). In response to the serum of treatment-naive patients, we recorded higher levels of LTB_4_ as compared with incubation with eculizumab-treated serum or PBS. We also treated PMNs with the serum of treatment-naive patients as well as the C5aR inhibitor PMX53. In this setup, LTB_4_ levels were also reduced, suggesting that the interaction of C5a and C5aR is required for the effect of eculizumab on downstream products of ALOX5 metabolism.

Taken together, eculizumab is likely to prevent ligation of C5aR on PMNs by inhibiting the generation of C5a. C5aR activation is needed to induce ALOX5 processing of AA; thus, eculizumab may decrease the generation of downstream leukotrienes.

## Discussion

Eculizumab-mediated ablation of terminal complement activation has proved effective for treatment of anti-AChR Ab^+^ MG patients (3). In order to improve treatment outcomes, knowledge of the consequences of complement modulation in the human system might be of value.

To this end, we analyzed the proteometabolome in a cohort of eculizumab patients, observing distinct patterns of signaling pathways induced by terminal complement ablation. First, we were interested in dissecting eculizumab’s mechanism of action using a proteomics approach. Interestingly, eculizumab-mediated complement blockade is not without contestation, as previous immunoassays failed to detect reduced C5a levels in response to eculizumab treatment (25). This might be due to eculizumab inducing a C5a neopeptide falsely detected by immunoassays (26). Analysis of our proteomics data revealed an accumulation of C6 and C7 in serum of eculizumab-treated patients. These findings corroborate the mechanisms of action of eculizumab as eculizumab inhibits the cleavage of C5. Interestingly, levels of C8 and C9 remained unchanged using a proteomics approach. In this context, the influence of eculizumab on the peripheral level of terminal complement components remains heterogenous. In patients with atypical hemolytic uremic syndrome (27) or thrombotic microangiopathy after hematopoietic stem cell transplantation (28), eculizumab reduced sC5b-9 concentrations, while other studies did not observe a change in response to treatment (29). Indeed, eculizumab-mediated complement inhibition is dependent on pretreatment complement levels as well as on the underlying disease (30). Consequently, terminal complement patterns due to eculizumab treatment are likely subject to variation between diseases and individual patients.

We analyzed the serum proteome in order to elucidate the intricate relationship between complement modulation and cellular processes, observing alterations in the lipid metabolism; factors of innate immunity, e.g., TF; the oxidative stress response; and the heterogenous group of MAPK signaling pathways. Indeed, complement has been shown to induce MAPK signaling (31). As such, C5a-C5aR interaction contributes to renal injury by MAPK activation and release of pro-inflammatory cytokines in a trichloroethylene-sensitized mouse model (31, 32). Consistent with this, we observed alterations of proteins associated with MAPKs, e.g., upregulation of MAP3K8 and downregulation of SKAP2 or MAPK8. Interestingly, we also detected proteins with antioxidant function, e.g., PRDX2 and PRDX6, to be abundant in serum of eculizumab-treated patients. Complement activity contributes to tissue injury in response to oxidative stress with the classical and the lectin — but not the alternative — pathways mediating complement activation (33, 34). In contrast, little is known about the retrograde influence of complement inhibition on the oxidative stress response. Our data point to a potentially beneficial influence of eculizumab on the oxidative stress response.

Intriguingly, we also observed metabolic alterations in eculizumab-treated patients. AA metabolism is localized at the intersection point of cardiovascular and inflammatory biology (35, 36). Three distinct enzyme systems metabolize AA, resulting in a plethora of biological effects. First, cyclooxygenases generate prostanoids, such as thromboxane A_2_ or prostaglandins, from AA. Second, the cytochrome P450 system metabolizes AA, generating hydroxyeicosatetraenoic acids capable of mediating pro-inflammatory signals (37). Last, the lipoxygenase pathway results in the formation of leukotrienes (38). ALOX5, perhaps the most studied lipoxygenase, inserts oxygen at the C-5 position of AA to promote the formation of 5-HPETE and finally LTA_4_, the precursor of downstream leukotrienes (17). The biological relevance of this pathway is underlined by the efficacy of leukotriene antagonists, and ALOX5 inhibitors are used to treat bronchial asthma and seasonal allergies (38, 39). Of note, C5a has been demonstrated to recruit ALOX5 to process AA and stimulate leukotriene metabolism (40). Concurrently, liberation of thromboxane B2 and other metabolic products of AA degradation increases in the presence of complement proteins (41, 42). Following this line of argument, proteomic and metabolomic analyses corroborated that AA metabolism was blunted in response to eculizumab as 5-HPETE and LTA_4_ — downstream products of AA processing by ALOX5 — were reduced compared with treatment-naive or azathioprine-treated patients. As an immunometabolic regulator, ALOX5 is primarily expressed by immune cells, such as granulocytes, macrophages/monocytes, and dendritic cells (43). Leukotrienes resulting from AA metabolism exert chemotactic properties and regulate immune cell activity and bronchoconstriction (43, 44). On a mechanistic level, C5a is required for ALOX5-mediated leukotriene generation by interacting with C5aR. Alterations of ALOX5 and downstream metabolites indicate the modulation of AA by terminal complement ablation as a potential off-target effect of eculizumab. Further studies are needed to understand whether modulation of leukotriene metabolism is indeed beneficial and to what extent this effect contributes to the clinical efficacy of eculizumab.

Given the rarity of MG and further aggravation by the limited application of eculizumab in clinical settings, this study is confined to a sample size of 10 patients for the eculizumab group. However, this study is designed as an explorative approach to better understand complement-modulating therapies. Consistent with this, the scope of an omics approach in a human model is not suited to deduce causality, but rather to highlight understudied or unknown mechanisms and stimulate further research. Further, assessment of metabolite levels is influenced by a plethora of confounders, including nutritional or environmental factors. Accordingly, additional studies are needed to account for potential external factors influencing metabolite readouts.

Nonetheless, these findings demonstrate that the eculizumab-induced proteometabolome is a valuable model for understanding the influence of terminal complement ablation on human biology and for exploring potential mechanisms shaping treatment outcomes beyond inhibition of the complement cascade.

## Methods

### Ethics and human participants.

All patients were required to meet the national guidelines for the diagnosis of MG (45). We included 3 age- and sex-matched cohorts with a total of 30 patients for cross-sectional comparison (*n* = 10 per group). Clinical and demographic baseline data are given in Table 1. We compared eculizumab patients with azathioprine (as a prominent first-line therapy) and treatment-naive patients. Patients were required to be treated with eculizumab for at least 3 months prior to blood sampling. To adjust for disease severity as a potential confounder, patients treated with eculizumab and patients treated with azathioprine were required to meet the following criteria defining a therapy-refractory course of disease in accordance with national guidelines (45): (a) Clinical severity: Myasthenia Gravis Foundation of America status of ≥III. (b) Previous immunotherapies: 2 previous immunotherapies with a treatment period of at least 12 months for azathioprine, mycophenolate, cyclosporine A, methotrexate, or steroids at maximum tolerable dose without sufficient symptom control. (c) Adverse effects: discontinuation of at least 2 previous immunotherapies due to intolerable adverse effects. (d) Myasthenic crisis: myasthenic crises requiring intensive care treatment.

For this study, patients treated with eculizumab and azathioprine were required to fulfil criterion (a) and at least 1 other criterion. Azathioprine-treated patients were required to be eligible for eculizumab treatment. Treatment-naive patients were required not to meet any of criteria (b) to (d). Both eculizumab- and azathioprine-treated patients were required not to be concurrently treated with a second IST. Low-dose steroids (<10 mg/d) were permitted.

### Biomaterial and patient cohort.

Serum samples were acquired after obtaining written informed consent. At the time of sampling, patients were required to have no evidence of infection. Serum samples from patients were stored according to the predefined standard operating procedure at the local biobanks of the Heinrich Heine University Düsseldorf and the Charité Berlin at –80°C. Later, they were transferred for measurement on dry ice to the Core Unit Proteomics facility of the University of Münster.

### Lysate generation and processing for proteomic deep mapping.

Serum samples (200 μL) were depleted according to the instructions of the manufacturer using the ProteoMiner kit (Bio-Rad Laboratories Inc.). This subproteome was placed in Pall Nanosep 10K Omega filter units (10 kDa cutoff) and centrifuged (12,500*g*, room temperature, 30 minutes). The analyte was washed by adding 100 μL of urea buffer (8 M urea, 100 mM Tris base) to the filter unit and was centrifuged (room temperature, 200*g*, and 30 minutes; same centrifuge settings used throughout). For reduction (45 minutes), 100 μL of 50 mM dithiothreitol in urea buffer was added to the filter unit. Subsequently, the unit was again centrifuged, and the sample was rinsed with 100 μL urea buffer. For alkylation, 50 mM iodoacetamide in urea buffer was placed into the filter unit. Incubation proceeded in the dark for 30 minutes at room temperature. Following centrifugation and rinsing twice with 300 μL 50 mM NH_4_HCO_3_ containing 10% acetonitrile in urea buffer and 200 μL of 0.01 μg/μL trypsin in 50 mM NH_4_HCO_3_ containing 10% acetonitrile were added to the filter unit. Incubation proceeded at 37°C overnight. Peptides were collected by rinsing the filter thrice with 5% acetonitrile/0.1% formic acid followed by centrifugation. Samples were dried using a Speedvac (Thermo Fisher Scientific) and redissolved in 10 μL 5% acetonitrile/0.1% formic acid.

### Mass spectrometry–based proteomics.

Peptide solutions (0.5 μL) were analyzed by reversed-phase chromatography coupled to ion mobility mass spectrometry with SYNAPT G2-Si/M-Class nano-UPLC (Waters Corporation) using PharmaFluidics C18 μPAC columns (trapping and 50 cm analytical). Data were analyzed using Progenesis for Proteomics (Waters Corporation) and the UniProt human database. One missed cleavage was allowed, carbamidomethylation was set as fixed, and methionine oxidation was set as a variable modification. A short list of the protein output was created by demanding protein assignment by at least 2 peptides. Histograms of the acquired proteome (Supplemental Figure 1) illustrate normal distribution of included patient samples.

### Lysate generation and processing for metabolomic profiling.

Serum (10 μL) was filtered using Pall Nanosep 10K Omega units (3.5 kDa cutoff), washed thrice with 5% acetonitrile/0.1% formic acid, dried, and redissolved in 10 μL 5% acetonitrile/0.1% formic acid.

### Mass spectrometry–based metabolomics.

Mass spectrometry was performed using above instrumentation in MSe mode. For data analysis we used Progenesis with Metascope, LipidBlast, ChemSpider, and Metlin databases. Hits with multiple substance assignments were not used. Metabolites were identified based on their mass, retention time, and mass spectra. A total of 8,893 hits were recorded. After metabolite extraction and exclusion of missing values, 289 unique metabolites were permitted for downstream analyses. Distribution of metabolites across patient samples is illustrated as histograms (Supplemental Figure 2).

### ELISAs.

The patients’ serum samples were tested protein levels via ELISA kits according to the manufacturer’s instructions. These included ALOX5 (Abbexa, catalog abx250809), C4BP (MyBioSource, catalog MBS9303010), C6 (Abcam, catalog ab230934), C7 (Abcam, catalog ab125964), CCL22 (Bio-Techne, catalog DMD00), LTA_4_ (Abbexa, catalog abx150355), LTB_4_ (Abbexa, catalog abx257172), and TF (Abcam, catalog ab220653). We performed dilution series and used dilutions of 1:50 for the final assays. Samples were measured in technical duplicates with the Tecan plate reader Infinite M200 Pro.

### Cell culture experiments.

PMNs were isolated from peripheral blood from healthy donors as previously described based on density gradients (22). PMNs were resuspended in DMEM in 6-well dishes with 10^6^ cells per sample. PMNs were incubated at 37°C and 5% CO_2_. PMNs were treated with substances at indicated concentrations or with serum samples for 6 hours. Afterward, supernatants were harvested and stored at −80°C. Concentration of LTB_4_ was determined by ELISA according to the manufacturer’s instructions. For stimulation with C5a, we used recombinant C5a (R&D Systems). PMX53 and zileuton were purchased from R&D Systems. For co-incubation with human serum, 500 mL of serum was added to each well containing 1 mL of DMEM.

### Visualization.

Figures were created using Adobe Illustrator (version 2020) and Servier Medical Art (https://smart.servier.com).

### Statistics.

Statistical analysis was performed using R 3.5.3. Data were presented as median (IQR), mean (SD), or *n* (%). Differences between groups were analyzed using unpaired Student’s *t* test or Mann-Whitney *U* test as appropriate. One-way ANOVA test or Kruskal-Wallis test was used for multiple groups as appropriate.

Prior to multivariate analysis, data were centered, and unit variance scaling was utilized. For PCA, each patient was treated as 1 data point. For heatmaps, rows and columns were clustered using correlation distance and average linkage. Raincloud plots were constructed as previously described (46). Volcano plots were created by plotting log_2_ values of the relative difference between the mean protein expression values against the –log_10_ *P* values. For simple regression analysis, we included the Quantitative MG score, MG Activities of Daily Living score and the MG Quality of Life score as dependent variables. sPLS-DA was performed on normalized metabolomics data using mixOmics package version 6.16 as previously described (47, 48) (Supplemental Figure 3A). The model was tuned using a 3-fold internal cross validation repeated 50 times to minimize the balanced error rate (Supplemental Figure 3B).

For enrichment analysis, differentially expressed proteins were defined as having an adjusted *P* value of < 0.05. We employed the following databases to identify the enriched MFs and associated biological pathways: GO (49), KEGG (50), and the HPA (51). The functional enrichment analysis for proteomics was performed using the R package gProfiler (version 0.70) with g:SCS multiple-testing correction method applying significance threshold of 0.05 (52). Functional enrichment of metabolite data was performed with the MetaboAnalyst R package version 3.0 set to GSEA as previously described (53). Differences were considered statistically significant with the following *P* values: *P < 0.05, **P < 0.01, ***P < 0.001, and ****P < 0.0001.

### Study approval.

All work performed in this study was in accordance with the Declaration of Helsinki. For this study, patients were recruited from the University Hospital Düsseldorf (Ethikkomission der HHU, Düsseldorf, Germany; ethics approval: 2016-053-f-S and 2021-1417) and the Charité Berlin (Ethikkomission der Charité Berlin, Berlin, Germany; ethics approval: EA1/281/10). Patients provided oral and written informed consent before inclusion.

### Data availability.

The full data set is available via ProteomeXchange with identifier PXD040786. The analytical code for the manuscript is available from the corresponding author on reasonable request.

## Author contributions

CBS, CN, SGM, and TR designed the study and methods; formal analysis was done by CBS and CN; CBS and CN performed the experiments and were responsible for data analysis; clinical data were provided by CBS, CN, FS, MP, AM, SGM, and TR; resources were provided by FS, AM, SGM, and TR; CBS and CN wrote the original draft; FS, NH, MP, AW, SR, NM, UD, ST, KS, AR, AM, SGM, and TR reviewed and edited the manuscript; figures were created by CBS, CN, and NH; funding acquisition was performed by SGM and TR; and supervision was provided by AM, SGM, and TR. CN and CBS contributed equally. The order was determined by coin flip.

## Supplementary Material

Supplemental data

## Figures and Tables

**Table 1:**
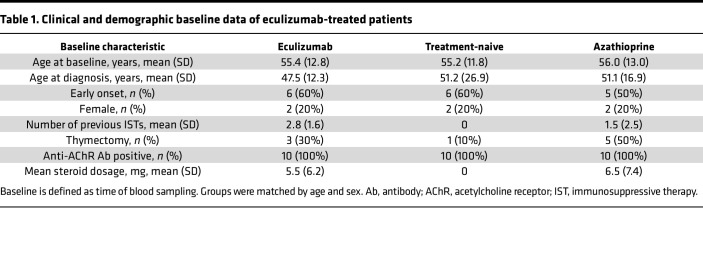
Clinical and demographic baseline data of eculizumab-treated patients

**Table 2:**
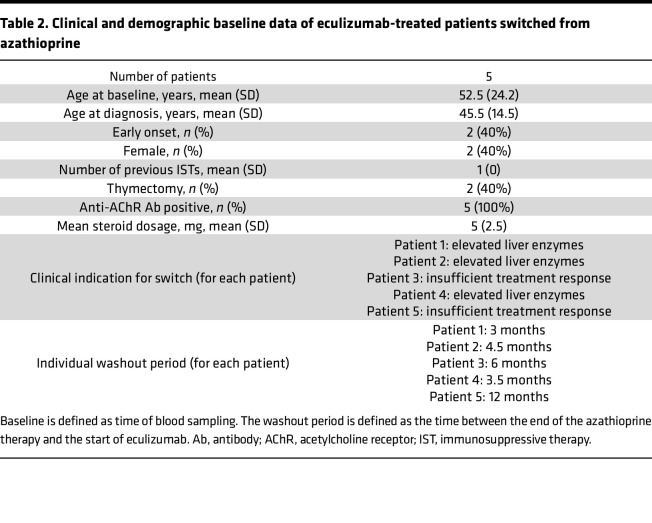
Clinical and demographic baseline data of eculizumab-treated patients switched from azathioprine

## Version 1 05/25/2023

In-Press Preview

## Version 2 07/10/2023

Electronic publication
