# Supplementary material for: Eculizumab treatment alters the proteometabolome beyond the inhibition of complement
Source: JCI Insight. 2023 Jul 10;8(13):e169135. doi: 10.1172/jci.insight.169135 (PMC10371336; doi:10.1172/jci.insight.169135)
Supplement: Supplemental data [file jciinsight-8-169135-s159.pdf]

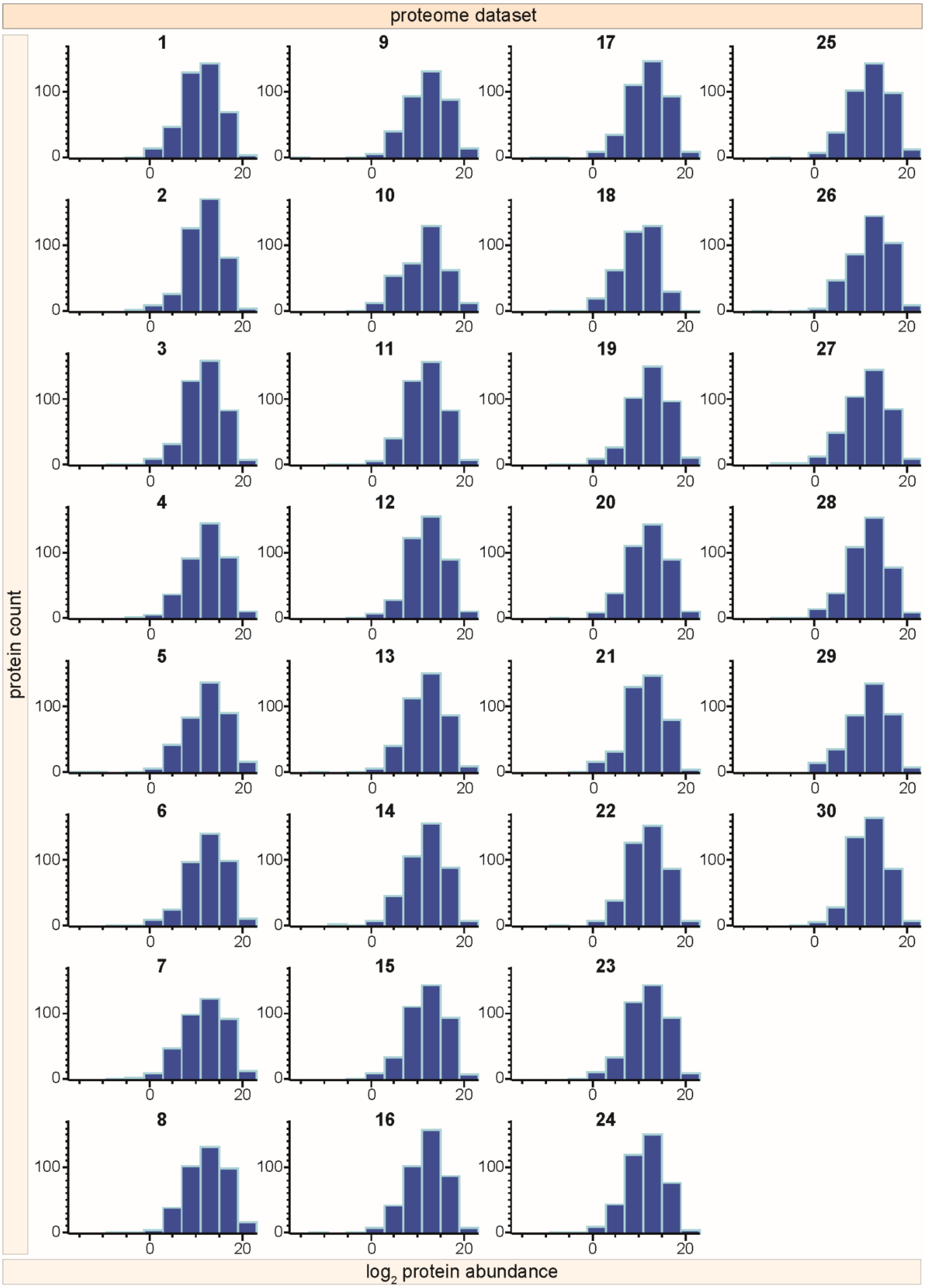

Supplemental Figure 1: Histograms of protein distribution across each patient's sample. Protein counts are depicted against the log2 value of protein intensities

illustrating protein numbers and abundance. Here, normal distribution of the proteome dataset is shown across all patients' samples.

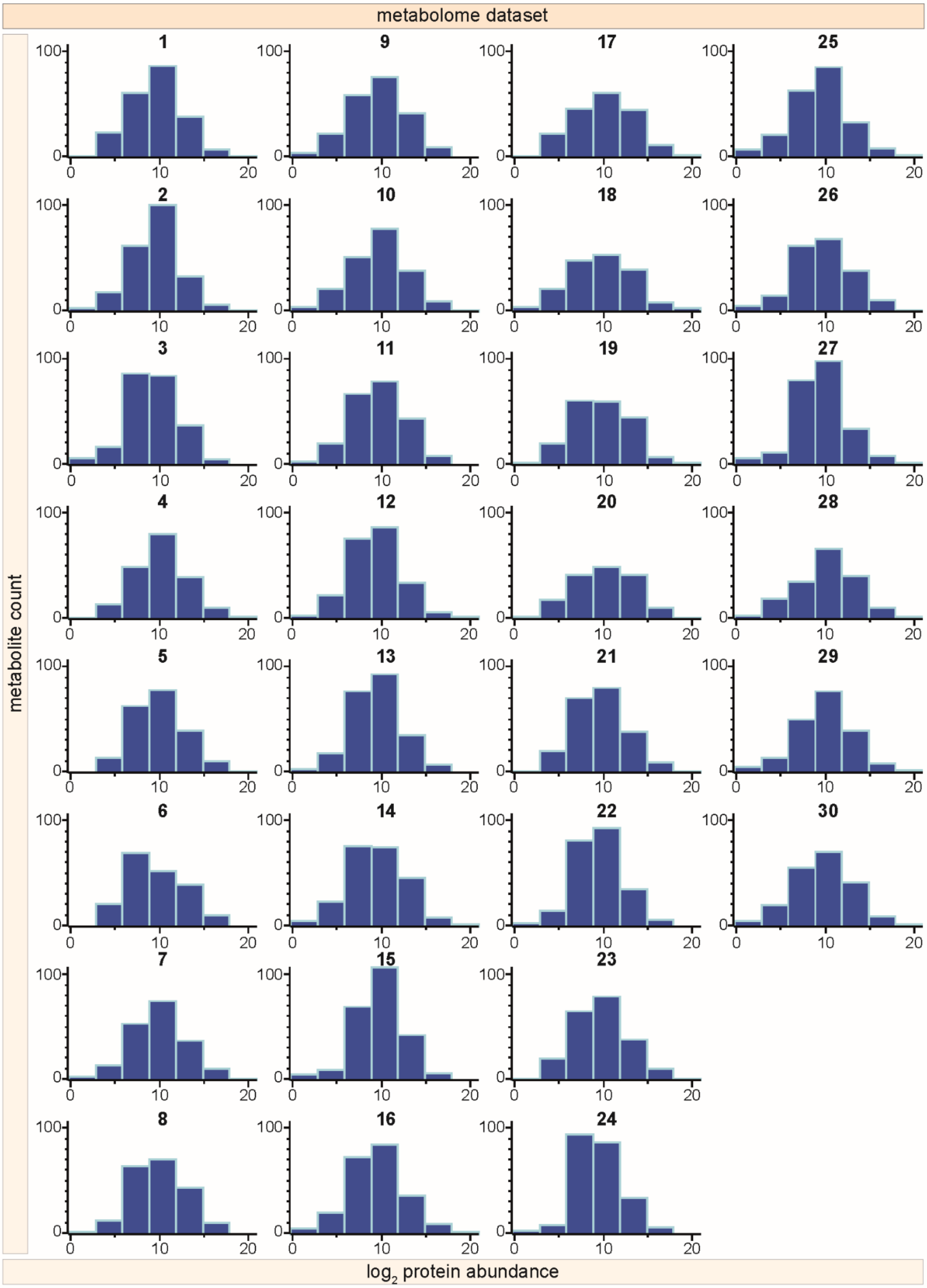

metabolome dataset
metabolite count
log2 protein abundance
1
2
3
4
5
6
7
8
9
10
11
12
13
14
15
16
17
18
19
20
21
22
23
24
25
26
27
28
29
30
100
0
10
20

Supplemental Figure 2: Histograms of metabolite distribution across each patient's sample. Metabolite counts are depicted against the log2 value of protein intensities illustrating protein numbers and abundance. Here, normal distribution of the proteome dataset is shown across all patients' samples.

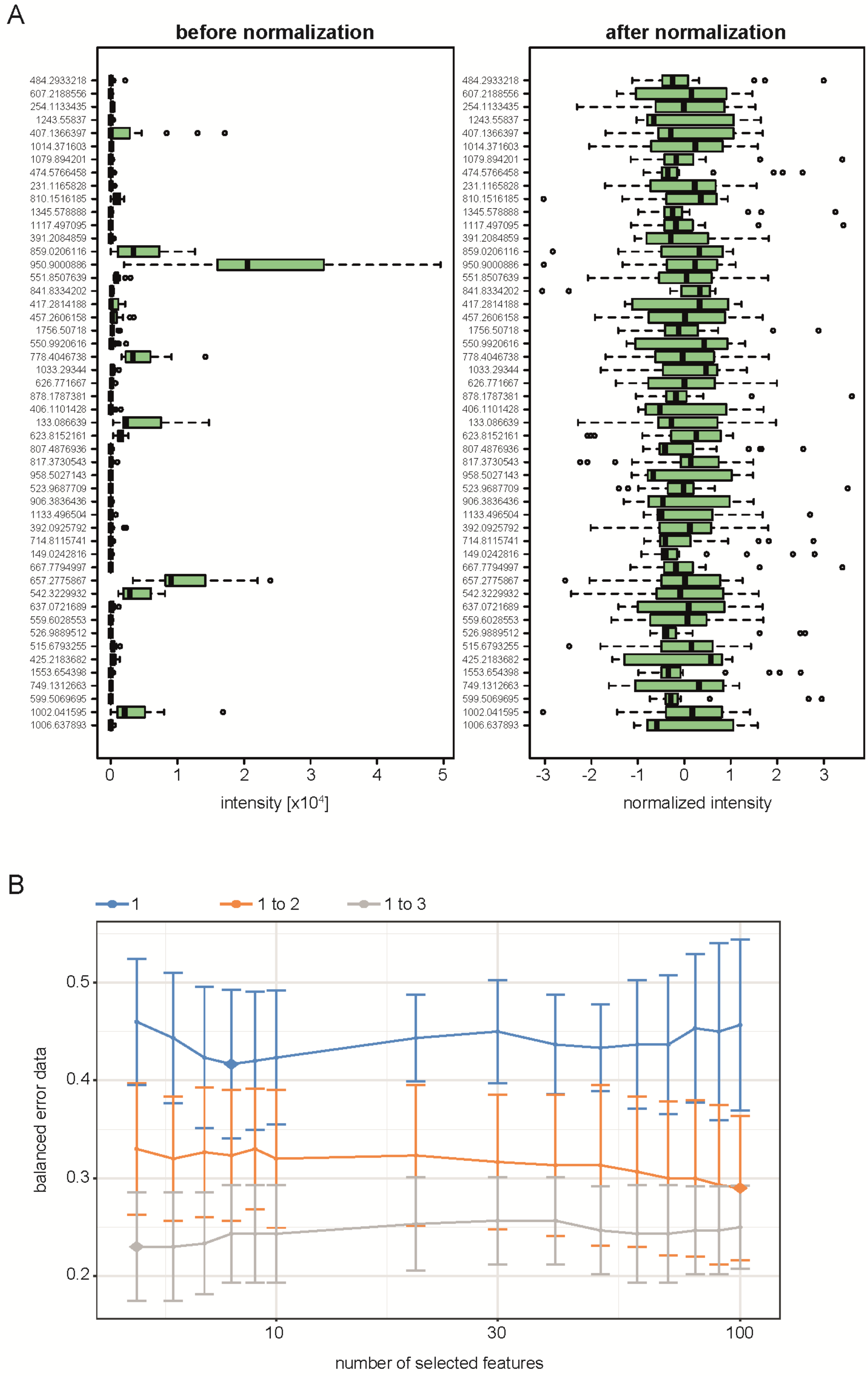

Supplemental Figure 3: (A) Exemplary MS/MS data before (left) and after (right) unit variance scaling. (**B**) Tuning of optimal number of variables for sPLS-DA. Variables

obtaining the lowest balanced error rate were used for each component. Abbreviations: sPLS-DA = Sparse Partial Least-Squares Discriminant Analysis.

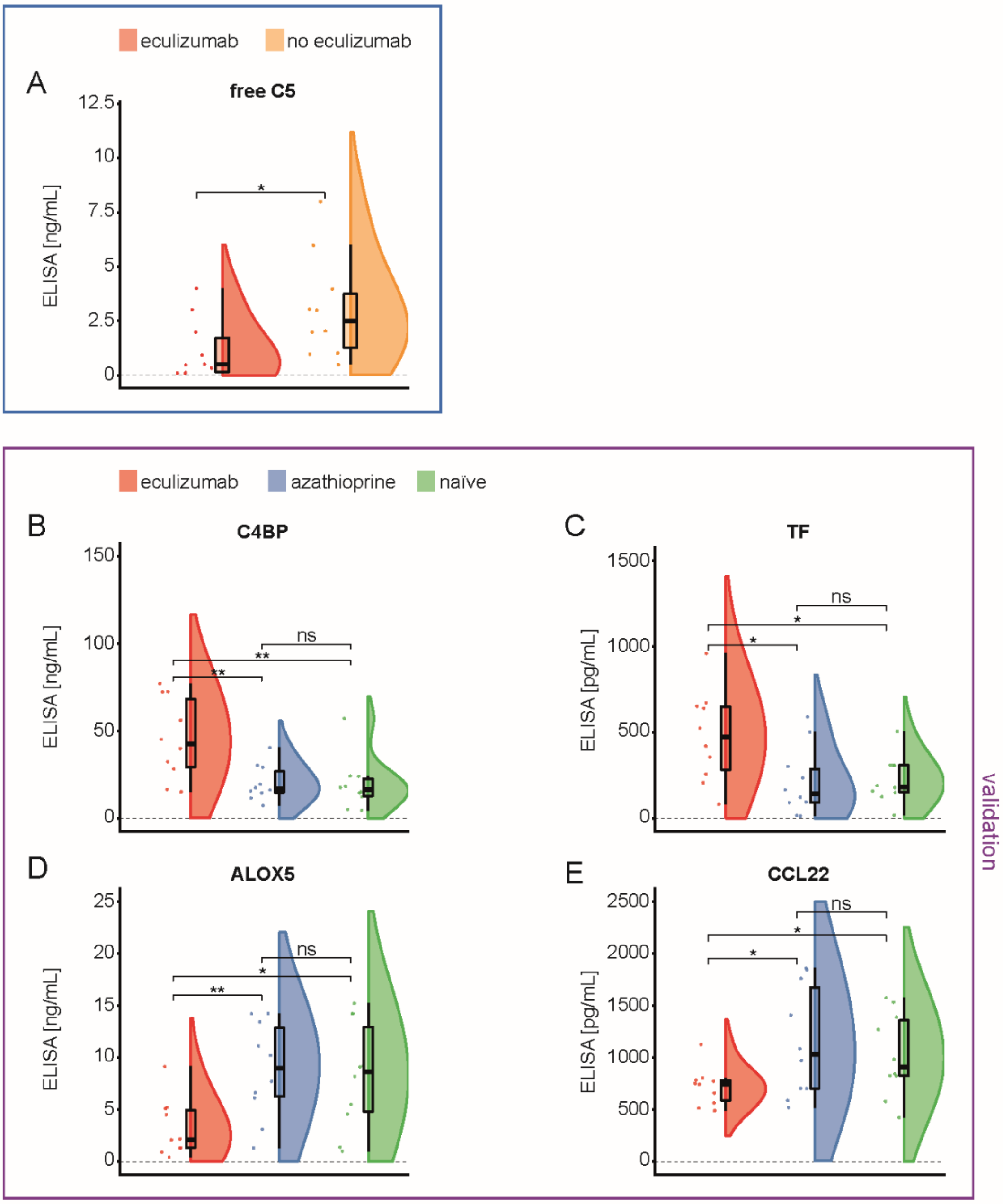

Supplemental Figure 4: (A – D): Protein abundance of regulated serum proteins of interest displayed as raincloud plots. Protein levels were determined by ELISA measurement. Whiskers are 1.5 IQR. To account for multiple comparisons, statistical significance was corrected by the false discovery rate (FDR) approach. A threshold of Q = 5% was used for FDR. We analysed 10 patients per group. Abbreviations: ALOX5 = arachidonat-5-

lipoxygenase, C4BP = C4b-binding protein, CCL22 = C-C motif chemokine ligand 22, IQR = interquartile range, TF = transferrin. $p < 0.01$ **, $p < 0.05$ *.
